# Auditory-conceptual associations in *Peter and the Wolf* and *Carnival of the Animals*: Evidence from 6- to 9-year-old children

**DOI:** 10.3758/s13423-025-02804-4

**Published:** 2026-01-05

**Authors:** Nicola Di Stefano, Alessandro Ansani, Valentina Focaroli, Rebecca Borsella, Giuditta Formenti, Andrea Velardi, Andrea Schiavio, Charles Spence

**Affiliations:** 1 Institute of Cognitive Sciences and Technologies (ISTC), National Research Council (CNR), Via Gian Domenico Romagnosi, 18A, 00196 Rome, Italy; 2 Centre of Excellence in Music, Mind, Body and Brain – Department of Music, Art and Culture Studies, University of Jyväskylä, Jyväskylä, Finland; 3 Department of Economic, Psychological, Communication, Education, and Motor Sciences “Niccolò Cusano” University, Rome, Italy; 4 Department of Political, Juridical, Sociological and Humanistic Sciences, “Niccolò Cusano” University, Rome, Italy; 5 School of Arts and Creative Technologies, University of York, York, UK; 6 Department of Experimental Psychology, University of Oxford, Oxford, UK

**Keywords:** *Carnival of the Animals*, Cross-modal associations, Music perception, *Peter and the Wolf*, Bayesian statistics

## Abstract

This study investigated auditory-conceptual associations in children using complex audiovisual stimuli, namely musical excerpts from the Western classical repertoire and drawings. In Experiment 1, we examined whether 6- to 9-year old children were able to consistently match musical excerpts from Prokofiev’s *Peter and the Wolf* with corresponding black-and-white images of the characters. The results confirmed robust associations, particularly for the bird, wolf and duck, while other pairings were more variable. In Experiment 2, we extended this approach by using the musical suite Saint Saëns’s *Carnival of the Animals*, testing whether timbre influences children’s audiovisual associations. Children were presented with colour images of animals alongside orchestral or piano versions of the musical excerpts that the composer associated with the animal. The results revealed that, in line with a similar study conducted recently in adults (Di Stefano et al., 2025), participants made significantly above-chance associations for the characters of the lion and the swan. However, unlike in adults, timbre had no significant effect on children’s audiovisual pairings. These findings highlight the robustness of auditory-semantic associations presented through audiovisual stimuli in childhood, supporting the idea that certain audiovisual correspondences are developmentally stable, while showing that subtle nuances (i.e., differences in timbre) might emerge later on during development.

**Supplementary Information:**

The online version contains supplementary material available at 10.3758/s13423-025-02804-4.

## Introduction

Cross-modal associations, also known as cross-modal correspondences, refer to the tendency for a sensory feature, attribute or dimension in one sensory modality – no matter whether physically present or merely imagined – to be systematically associated with a feature, attribute or dimension in another sensory modality (Motoki et al., 2023; Spence, 2011). For instance, auditory pitch has been consistently associated with both spatial elevation and object size (see Spence, 2019, for a review), brightness (e.g., Brunel et al., 2015; Klapetek et al., 2012; Marks, 1974, 1987), hue (e.g., Melara, 1989; see Spence & Di Stefano, 2022, for a review), and angularity (Marks, 1987; Parise & Spence, 2012). The research shows that pitch-based correspondences extend to other sensory modalities, such as touch, with lower-pitched sounds being associated with rough textures while higher-pitched sounds are associated with softness instead (e.g., Eitan & Timmers, 2010; Hamilton-Fletcher et al., 2018; see Di Stefano & Spence, 2022, for a review on roughness).

One of the central challenges in developmental psychology and cognitive neuroscience is understanding how children come to integrate information from different sensory modalities – a capacity that supports everything from object recognition to social cognition and language learning. Cross-modal associations may not merely reflect perceptual biases but could also reflect early-developing learning mechanisms that support the acquisition of symbolic knowledge (e.g., Bahrick & Lickliter, 2000; Smith, 1994; Walker-Andrews, 1994). In this sense, studying children’s ability to form reliable audiovisual associations offers insight into how conceptual representations are shaped across development. These correspondences may scaffold semantic learning (e.g., through metaphor or analogy) and may form the basis for later-developing symbolic or aesthetic reasoning.

Although audiovisual pairings have been the primary focus of researchers studying cross-modal correspondences (see Spence, 2011, for a review; Spence & Sathian, 2020, p. 239), relatively few empirical studies have examined how these associations develop across the lifespan, particularly in children (Fernández-Prieto et al., 2015; Meng et al., 2023; Mondloch & Maurer, 2004; Nava et al., 2016; Wallmark & Allen, 2020; Walker et al., 2010; see also Marks et al., 1987). The literature suggests that correspondences between basic sensory features, such as ascending/descending pitch and spatial height, are present in 3- to 4-month-old infants (Walker et al., 2010, see also Dolscheid et al., 2014). The pitch-size correspondence has been demonstrated in 6-month-old infants (Fernández-Prieto et al., 2015). Perceptual associations between size or brightness and pitch have been observed to emerge later on in the development at 30- to 36-month-old children (Mondloch & Maurer, 2004; see also Haryu & Kajikawa, 2012). By demonstrating audiovisual associations in infants, this body of literature supports the fundamental role of cross-modal perception in early development, shaping infants’ intersubjectivity and understanding of the world (e.g., Stern, 1985; see Meltzoff & Borton, 1979, for critical evidence on neonates).

While most studies have examined correspondences between simple sensory features or dimensions (e.g., pitch-size, pitch-shape, timbre-surface texture; cf. Spence & Di Stefano 2024), far less attention has been directed at auditory-conceptual associations involving more complex stimuli. One notable exception is the study by Trainor and Trehub (1992), which examined children’s ability to match representations of animals (wolf, bird, cat and duck) to corresponding musical excerpts from Prokofiev’s *Peter and the Wolf*. Their findings demonstrated that children, aged 4–6 years, matched the images to the musical pieces significantly better than chance, with the wolf and bird being more consistently associated than the cat and duck (see also Moore et al., 1999, on Saint-Saëns’ *Carnival of the Animals*).

To contribute to the limited research on cross-modal associations in children using complex audiovisual stimuli and to test, for the first time, the effect of timbre on the cross-modal pairings, we designed two experiments using both musical excerpts from Prokofiev’s *Peter and the Wolf* (Experiment 1) and Saint-Saëns’ *Carnival of the Animals* (Experiment 2). In Experiment 1, children aged 6–9 years were invited to listen to five musical excerpts from *Peter and the Wolf* and to choose one image that corresponded to the audio. Using a similar procedure, in Experiment 2, half of the participants were exposed to the excerpts from the *Carnival of the Animals* in the piano timbre, while the other half heard the orchestral version of the composition. After listening to the audio stimulus, participants were asked to choose one image that corresponded to the audio. Overall, the findings suggest that children’s audiovisual associations are not only internally consistent but mostly align with the patterns observed in adults (Di Stefano et al., 2024, 2025).

## Methods

### Experiment 1: Peter and the Wolf

#### Participants

The study was conducted at the Collegio della Guastalla primary school in Monza (Italy). The participants were children aged 6–9 years, attending first, second, and third grade of primary school. A total of 40 children took part in Experiment 1 (*M*_age_ 7.52 years, *SD* = 0.86, F = 13). Thirteen children were from first grade, 12 from second, and 14 from third. All participants were Italians, except for one Romanian girl. No child had any visual or auditory disabilities. The protocol was approved by the Research Ethics and Integrity Committee of the National Research Council of Italy (n. 0323801).

Along with giving consent, parents were invited to complete a questionnaire providing the following information: the child’s age, their profession, whether they were musicians or had musical expertise, and, if so, whether they played their instrument at home in the presence of their child. Additionally, they were asked about the number of children in the family and whether any of them attended music courses. These questions were aimed to assess the child’s exposure to musical stimuli throughout their life and to what extent they had developed a trained musical ear.

#### Stimuli

Both visual and auditory stimuli were the same as those used in Di Stefano and colleagues (2024). Visual stimuli consisted of black and white drawings of the following characters: the bird, the duck, the wolf, the grandfather and the cat (see Online Supplementary Materials (OSM), Fig. S4). The auditory stimuli consisted of the musical excerpts by Prokofiev associated with the animals. The musical excerpts were played on a Lenovo T470s, while visual stimuli were presented using sheets of paper.

#### Experimental procedure

Children were tested individually on different days. All of the tests took place in the morning, during regular school hours. After sitting in front of the experimenter, the children were greeted with some general questions, such as: "What are you doing in class?", "Do you play any instruments?", "What is your favourite subject?", with the goal of making them feel comfortable. Then, the experimenter would ask the children to recognise the images on the sheet and explain what they had to do using a general phrase like "Now you will listen to some music and have to match it to one of the images in front of you". The first music excerpt was then played, and the children chose the corresponding image. The piece could be replayed multiple times. After selecting the image, the next excerpt was played, continuing until all five pieces were completed. The order of presentation of both the auditory and visual stimuli was randomised for each participant, thus ensuring that the stimuli were presented in a different order for each child.

#### Statistical analysis

All of the analyses were run in the R environment (RStudio 2024.09.0 375 “Cranberry Hibiscus” for macOS). Given the multinomial nature of our dependent variable (i.e., the participant choice for every musical excerpt) and the repeated-measures design, a Mixed Multinomial Logit Model (MMNL: McFadden & Train, 2000) was implemented in a Bayesian framework through *brms* (Bürkner, 2017, 2018). All other indexes were computed through *modelbased* (Makowski et al., 2025) and *bayestestR* (Makowski et al., 2019a). The Mixed Multinomial Logit Model (MMNL) extends the standard multinomial logit model by allowing for individual-level variability in decision making. Specifically, unobserved heterogeneity (differences that are not directly measured) is captured by treating certain parameters (the baseline log-odds of choosing a certain image) as randomly distributed across individuals rather than fixed. This is particularly beneficial when dealing with repeated-measures designs, where each participant provides multiple responses, because the model can account for consistent yet idiosyncratic patterns in how each participant responds. Unlike a classic frequentist model, a Bayesian MMNL incorporates prior distributions and yields a posterior distribution of parameters, allowing us to make direct probability statements about the likelihood of the effects and providing a more nuanced treatment of uncertainty. A Hamiltonian Monte Carlo (HMC) method with a No-U-Turn Sampler (NUTS) algorithm was implemented to estimate Bayesian model coefficients: we ran six chains, each having 8,000 iterations, with a burn-in of 2,000. All models reached convergence as per the R-hat index (i.e., Rhat = 1). The initial formula, in Wilkinson notation, was:

$$\text{Choice }\sim \text{ Musical excerpt }+\left(1+\text{Musical excerpt}|\mathrm{Participant}\right)$$

Furthermore, as we were interested in how age impacts the accuracy of the associations, we fit two Bayesian Mixed Logistic models with a dichotomous variable as the dependent (i.e., accuracy) and musical excerpt and age as the predictors.

##### Priors and ROPE

In the Bayesian context, all model parameters are random variables, thus, we needed to assign priors. To reflect our neutral stance for the intercepts and regression coefficients, in line with Gelman and colleagues (2008), we used a zero-centred Cauchy distribution with scale 2.5. As for the variance parameters of the models, we used an exponential distribution with λ = 1 (Heiss, 2023; McElreath, 2020). These priors are considered weakly informative and have the advantage of letting the data speak with no strong preconceptions.

In our studies, to assess the associations, we first set a Region of Practical Equivalence (ROPE: Makowski, 2019b), namely, an area of the posterior parameter space wherein the predicted probabilities are so close to the chance-level point that such deviations are considered practically negligible (or indistinguishable from chance). In Experiment 1, we set the ROPE from 15% to 25%, the chance level being 20%. In Experiment 2, the ROPE was proportionally scaled to match that of Experiment 1, resulting in bounds of 10.71% and 17.85%, with a chance level of 14.28%. After fitting the Bayesian MMNL, we extracted posterior draws for all associations and applied the HDI+ROPE decision rule (Kruschke, 2018); that is, we assessed how much of the 89% Highest Density Interval (HDI) fell within the ROPE. If less than 2.5% of the HDI overlapped with the ROPE, we interpreted this as extreme evidence that the association deviated meaningfully from chance. If this proportion was between 2.5% and 5%, we considered this as strong evidence.

bIn the results, the reader will find the proportion of the 89% HDI inside the ROPE (*ROPE%*) and the Probability (*P*) together with its 89% credible intervals (Kruschke, 2015; McElreath, 2020). In all cases, the Maximum A Posteriori (MAP) was used as the central tendency measure (Makowski, 2019b).

### Results

**Fig. 1:**
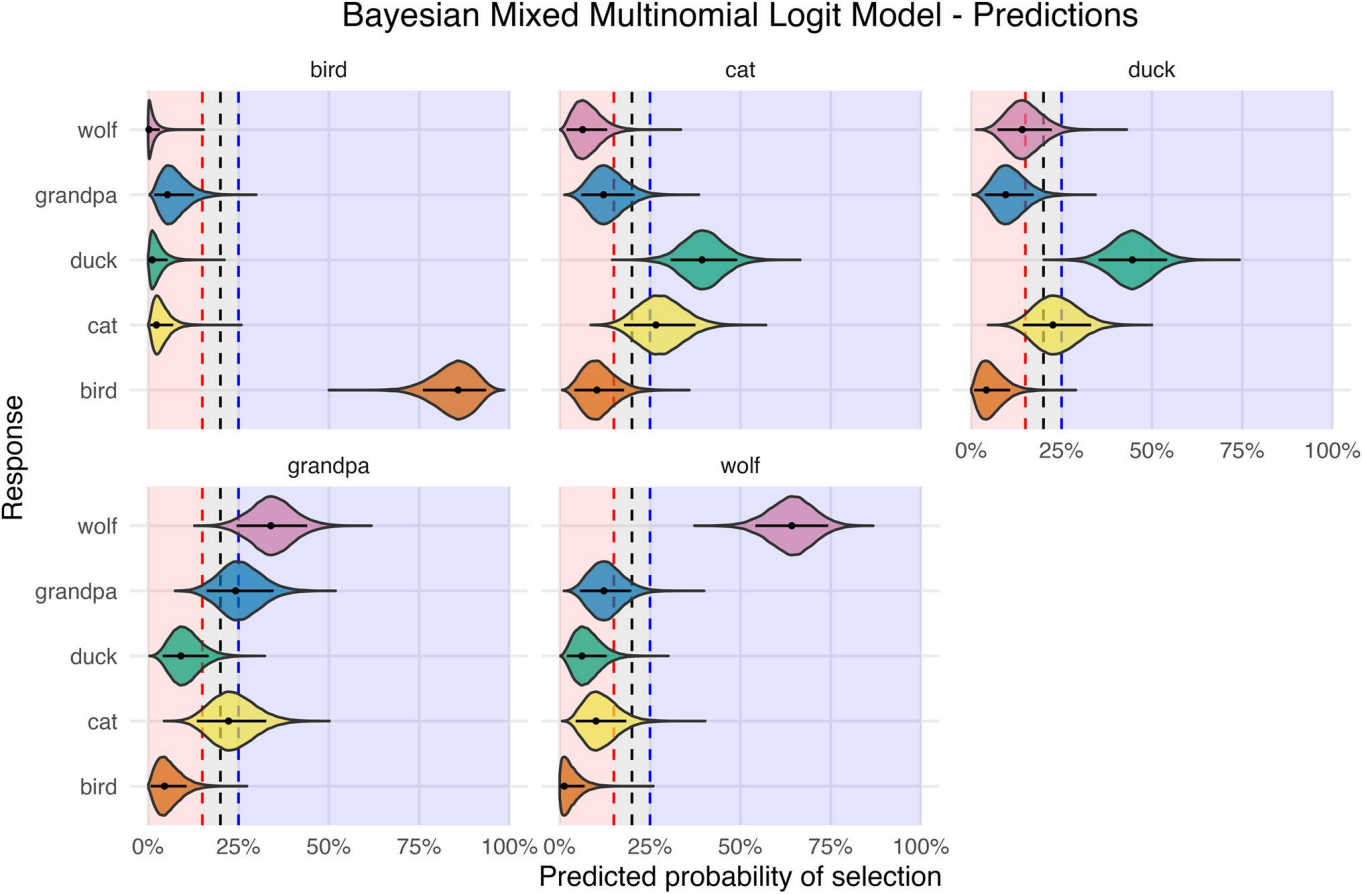
Predictions of the Bayesian Mixed Multinomial Logit Model. The label on top of each panel represents the musical excerpts. On the x-axis, the posterior distributions of the predicted probabilities of the image choices are plotted. On the y-axis are the participants’ responses (i.e., images). The area delimited by the red and blue dashed lines represents the ROPE. The horizontal black lines represent the 89% credible interval, whereas the middle point indicates the MAP

The predicted probabilities are represented in Fig. 1 and reported in Table S1 (OSM) along with a confusion matrix (see Fig. S1, OSM). At first glance, we realise that the variability in the estimated probability for the bird and wolf excerpts is way smaller than for all other musical themes. This result is consistent with the findings of Di Stefano and colleagues (2024), where the bird and wolf were by far the most consistent associations. In what follows, we report a more detailed description of the associations.

#### Bird

In the bird excerpt, the 89% HDI intervals of all images were fully outside the ROPE (*ROPE%* = 0). The probability of choosing the bird image was extremely high (*P* = 85.76%, 89% CI [76.01, 93.53]), whereas all other probabilities were below 6%.

#### Cat

In the cat excerpt, two associations were completely outside the ROPE, namely, the duck and the wolf. The former was the most chosen one (*P* = 39.39%, 89% CI [30.68, 49.13]), whereas the latter was selected very seldom (*P* = 6.34%, 89% CI [1.88, 13.13]). A large portion of the correct association’s HDI interval (*ROPE%* = 30.01) fell into the ROPE; as such, we consider this association not meaningfully different from the chance level. The associations with the grandpa (*ROPE%* = 31.82) and the bird (*ROPE%* = 13.86) were also inconclusive.

#### Duck

The correct association was clearly recognised, with a predicted probability more than twice the chance level. Conversely, the bird image was negatively associated with this excerpt (*P* = 4.18%, 89% CI [0.79, 10.76], *ROPE%* = 0). The remaining associations were likely due to chance (*ROPE%*_*cat*_ = 60.51; *ROPE%*_*wolf*_ = 45.89; *ROPE%*_*grandpa*_ = 12.84).

#### Grandpa

The grandpa excerpt was associated with the wolf image (*P* = 33.96%, 89% CI [24.48, 44.02], *ROPE%* = 0.04) and negatively associated with the bird (*P* = 4.53%, 89% CI [0.66, 10.64], *ROPE%* = 0). Large portions of the grandpa (*ROPE%* = 47.36) and cat (*ROPE%* = 61.15) images fell into the ROPE. As for the association with the duck, more than 90% of the HDI interval was below the ROPE region, thus suggesting a negative association, although with moderate evidence.

#### Wolf

Finally, similar to the bird excerpt, the wolf was exceptionally well associated with its image (*P* = 64.22%, 89% CI [54.16, 74.30], *ROPE%* = 0). The associations with the bird (*P* = 1.26%, 89% CI [0.00, 6.89], *ROPE%* = 0) and duck (*P* = 6.14%, 89% CI [0.01, 13.00], *ROPE%* = 0) were clearly negative. The associations with the cat (*ROPE%* = 18.24) and grandpa (*ROPE%* = 28.45) were not different from chance.

#### Age

Participant age was computed as a continuous variable by converting years, months, and days into a decimal number. Specifically, age was calculated as:

$$age = years + \frac{months}{12}+\frac{days}{365.25}$$

when inspecting the logistic model, interestingly, 98.06% of the distribution of the age parameter was negative; more precisely, its central tendency was log odds −0.53, 89% CI [−1.10, −0.13] (Cohen’s *d* ~ .29). In the Bayesian framework, this constitutes evidence that the effect is likely to exist. As an additional step to grasp its meaningfulness, we set a ROPE between log odds ± 0.18 (i.e., Cohen’s *d* ± 0.10) and found that 5.93% of the age parameter distribution lies in this range. Finally, when comparing the alternative hypothesis that the parameter is negative and meaningful (i.e., log-odds < −0.18) against the null hypothesis that it lies within the ROPE (Morey & Rouder, 2011), we obtained a Bayes Factor of 0.74, that is, anecdotal evidence against the presence of a meaningful negative effect.

### Discussion

The results of Experiment 1 indicate that participants consistently associated the music with the images of the wolf and bird in alignment with Prokofiev’s intentions. Beyond confirming previous findings in children (Trainor & Trehub, 1992), our study offers a more detailed analysis of these associations. The bird excerpt was overwhelmingly matched with the bird image, with all other associations falling well below chance level.^1^ Similarly, the wolf excerpt was strongly associated with the correct image, while incorrect pairings were systematically ruled out.

Notably, unlike adults (Di Stefano et al., 2024), children were also more likely to associate the excerpt of the duck with its corresponding image, though this effect was weaker as compared to the wolf and bird. In contrast, the music for the cat was consistently misattributed to the duck, while its correct pairing remained near chance level. It might be worth observing here that the themes of both the cat and the duck are played by woodwinds (clarinet and oboe, respectively). While the cat’s theme is slower and more sinuous, with some staccato touches that suggest stealth, the duck’s theme is lighter and more playful. The evident similarities between the two themes might thus partially account for participants’ misattribution of the cat’s melody to the image of the duck.

Regarding the misattribution of the music for the grandpa to the wolf, one might cautiously observe that, while the two musical themes differ significantly in contour and character, they share certain acoustic and timbral features that may contribute to the confusion. Both are situated in the lower register and are assigned to instruments with dark, resonant timbres – the bassoon for the grandfather and French horns for the wolf. Furthermore, the two characters may exhibit a degree of semantic overlap: each embodies traits associated with strength, authority, and a form of mature or imposing masculinity, which could reinforce the perceptual similarity despite their musical distinction.

Finally, our findings clearly demonstrate that age had a significant effect on children’s associations, indicating that accuracy decreased as children’s age increased. However, the evidence against the effect being larger than negligible is only anecdotal.

## Experiment 2: Carnival of the Animals

### Participants

Participants were the same as Experiment 1.

#### Stimuli

Visual stimuli consisted of coloured images of the following characters: lion, cuckoo, turtle, swan, elephant, donkey and chicken (see OSM, Fig. S5). The auditory stimuli consisted of the musical excerpts that Saint Saëns associated with each animal. The musical excerpts were played on a MacBook Air M1, while visual stimuli were presented using paper sheets. The visual and auditory stimuli were the same as used in Di Stefano and colleagues (2025).

#### Experimental procedure

The experimental procedure was similar to Experiment 1, except that half of the participants were exposed to the musical stimuli in the piano timbre, while the other half heard the orchestral version of the composition.

#### Statistical analysis

The analytic procedure was similar to Experiment 1. We thus immediately compared a model where the image was predicted by the melody against a model where we added an interaction term musical excerpt × timbre, namely:

$$\begin{array}{l}\text{Choice }\sim \text{ Musical excerpt }+\left(1+\text{ Musical excerpt }|\text{ Participant}\right)\\ \text{Choice }\sim \text{ Musical excerpt }\times \text{ Timbre }+ (1 +\text{ Musical excerpt }|\text{ Participant})\end{array}$$

### Results

**Fig. 2:**
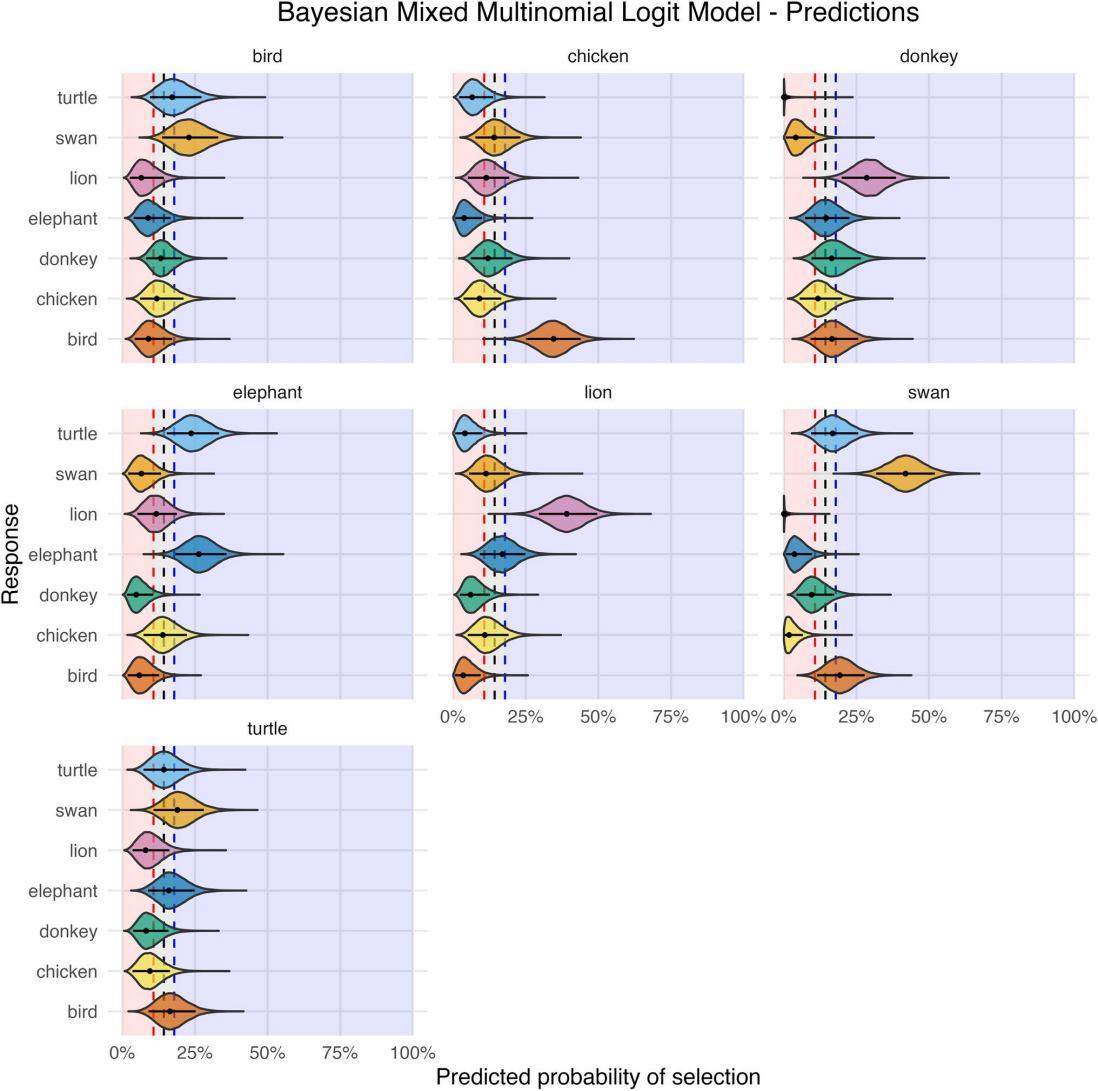
Predictions of the Bayesian Mixed Multinomial Logit Model. The label on top of each panel represents the musical excerpts. On the x-axis, the posterior distributions of the predicted probabilities of the image choices are plotted. On the y-axis are the participants’ responses (i.e., images). The area delineated by the red and blue dashed lines represents the ROPE. The horizontal black lines represent the 89% credible interval, whereas the middle point indicates the MAP

The two models were compared via leave-one-out cross-validation (LOO), WAIC and Bayes Factor (*BF*). The results of the LOO showed that the model with the timbre performed worse than the simpler model. The difference in expected log-predictive density (ELPD) was substantial (ΔELPD = 21.2, *SE* = 5.7)^2^ (Sivula et al., 2023). Similarly, the WAIC was better for the model without the timbre (ΔWAIC_simple_ = 1066.5, *SE* = 20.8; WAIC_timbre_ = 1103.1, *SE* = 23.8). When computing the *BF*, the evidence in favour of the first model over the second one was extreme (*BF* = 1.52 × 10^18^). These findings strongly indicate that the timbre did not play any role in shaping the response pattern of the participants (see also Fig. S2, OSM). As such, we use the simpler model to predict the probabilities. These are represented in Fig. 2 and reported in Table S2 (OSM), together with the confusion matrix (see Fig. S3, OSM).

#### Bird (Cuckoo)

In the cuckoo musical excerpt, none of the image associations were meaningfully far from the chance region (*ROPE%* ranging from 23% to 46%). The one with the lowest proportion in the ROPE was the swan, although with scarce evidence (*P* = 22.89%, 89% CI [13.65, 32.97], *ROPE%* = 13.99).

#### Chicken

In the chicken excerpt, the only image overcoming chance level was the bird (*P* = 34.59%, 89% CI [25.06, 43.92], *ROPE%* = 0), while the elephant was very distant from the chance level on the negative side (*P* = 3.77%, 89% CI [0.72, 9.97], *ROPE%* = 1.90%). As for the correct image, 41.82% of its HDI was within the ROPE.

#### Donkey

We found strong evidence that the donkey excerpt was associated with the lion, with 100% of the HDI lying above the ROPE (*P* = 28.52%, 89% CI [19.86, 38.58]). Moreover, the evidence for its negative association with the turtle image was extreme (*P* = 0.00%, 89% CI [0, 2.25], *ROPE%* = 0). We also found that the swan image had a strong negative association (*P* = 4.11%, 89% CI [0.06, 10.58], *ROPE%* = 4.61%). All other associations had more than 49% of their HDIs in the ROPE.

#### Elephant

The correct association was well recognised (*P* = 26.29%, 89% CI [18.16, 35.86], *ROPE%* = 0); although the turtle image was chosen only slightly less often (*P* = 23.67%, 89% CI [15.27, 33.28], *ROPE%* = 6.44). We also observed a negative association with the donkey (*P* = 4.73%, 89% CI [1.51, 10.62], *ROPE%* = 3.93).

#### Lion

We found substantial evidence that the lion excerpt was correctly associated with its image (*P* = 39.07%, 89% CI [29.50, 49.63], *ROPE%* = 0). Some of the other associations were very far from chance level and close to zero, especially the bird (*P* = 3.47%, 89% CI [0.05, 9.46], *ROPE%* = 0.74) and the turtle (*P* = 4.05%, 89% CI [0.07, 10.10], *ROPE%* = 2.42).

#### Swan

The swan excerpt was associated correctly with its image (*P* = 41.91%, 89% CI [31.75, 51.99], *ROPE%* = 0) with extreme evidence. Also in this case, we noticed that some of the associations were very close to zero, especially the lion (*P* = 0%, 89% CI [0, 2.20], *ROPE%* = 0), the chicken (*P* = 1.78%, 89% CI [0, 6.54], *ROPE%* = 0) and the elephant (*P* = 3.64%, 89% CI [0.65, 9.70], *ROPE%* = 1.48).

#### Turtle

All of the associations with the turtle excerpt were not far from the chance level, with ROPE% ranging from 34% to 59%.

#### Age

The same effect of age was found in Experiment 2. In this experiment, 98.96% of the distribution of the age parameter was negative; its central tendency was log odds −0.52, 89% CI [−0.92, −0.17] (Cohen’s *d* ~.28). In this case, 3.71% of the age parameter distribution lies in the ROPE. When comparing the alternative hypothesis that the parameter is negative and meaningful (i.e., log odds < −0.18) against the null hypothesis that it falls in the ROPE, we obtain *BF* = 0.89, namely, again anecdotal evidence against the effect being larger than negligible.

### Discussion

The results of Experiment 2 indicate that timbre did not significantly influence participants’ response patterns, suggesting that other musical features played a more prominent role in guiding the children’s associations. The swan excerpt was the most robustly identified, with strong evidence for its correct pairing. Similarly, the lion and elephant excerpts were associated with their correct images, while certain incorrect pairings, such as the lion excerpt with the bird and the turtle images, were strongly ruled out. Notably, the donkey and chicken excerpts exhibited unexpected associations, with the lion and bird being the most frequently selected images, while their correct counterparts remained largely unrecognised. The age of the children also played a role in shaping accuracy in Experiment 2, with older children demonstrating slightly lower accuracy levels, with evidence for this effect being similar to that in Experiment 1.

## General discussion

Taken together, the findings of this study demonstrate that children’s audiovisual associations are not only internally consistent but also broadly align with patterns of results that have recently been observed in adults (Di Stefano et al., 2024, 2025). These results contribute to broader theories of cross-modal learning and development by showing that children can reliably form associations between complex, semantically rich auditory and visual stimuli – associations that largely mirror those observed across cultures in adults. This supports the view that (certain) cross-modal correspondences are grounded in conceptual and affective mappings that remain stable throughout development (Motoki et al., 2023; Spence, 2011). Furthermore, these findings align with literature suggesting that cross-modal associations may serve as a scaffold for more abstract forms of cognition, including metaphorical thinking, emotional interpretation, and symbolic understanding (Lakoff & Johnson, 1980; Marks, 1987; Wallmark & Allen, 2020). The ability to match music with visual representations of characters may therefore reflect not only low-level perceptual processing but also children’s emerging capacity for conceptual inference and the decoding of emotional meaning across modalities.

Besides aligning with Trainor and Trehub’s (1992) findings, the results of Experiment 1 align with those obtained from adults by Di Stefano and colleagues (2024). In that study, the protocol allowed for conclusions regarding the role of emotional mediation in driving the cross-modal associations. Given the similarity of the results, we can hypothesise that a similar mechanism influenced children’s associations, with the music linked to the wolf and the bird representing contrasting features – both musically (e.g., major vs. minor, bright vs. dark timbre, slow vs. fast tempo) and affectively (e.g., joyful vs. aggressive, light vs. dark). Thus, children may have been guided by this polarity in terms of their matchings (e.g., Smith & Sera, 1992).

The findings of Experiment 2 suggest that, unlike adults (see Di Stefano et al., 2025), children’s associations were not influenced by timbre. Their responses remained consistent across both groups – those who listened to *Carnival of the Animals* in the orchestral version and those who heard the piano version. These results might be surprising, given empirical evidence showing that even younger children (3–5 years old) exhibit greater sensitivity to timbre than to pitch contour (Creel, 2016, 2023). However, our findings simply indicate that timbre did not influence their performance in the cross-modal association task, with no direct implication for sensitivity to timbre or timbre discrimination. This aligns with the findings reported by Wallmark and Allen (2020), who investigated preschoolers’ (ages 3–6 years) cross-modal mappings of timbre onto touch and vision. In that study, a significant main effect of age on cross-modal congruency indicated that timbral brightness mappings evolve throughout early childhood and stabilise only later. Therefore, other features of the musical stimuli, such as pitch contour, harmony, and rhythmic profile, might have driven children’s pairings more than timbral quality. In future, studies could address this issue by exploring whether explicitly directing children’s attention to timbre – through training or priming – would enhance its role in audiovisual pairings.^3^

In both Experiment 1 and Experiment 2, the age of the child had an effect, with accuracy in audiovisual pairings decreasing as their age increased. This effect suggests a developmental shift in how children process audiovisual associations. From a developmental perspective, the fact that younger children showed more consistent associations might reflect a stage where perceptual-affective mappings are more salient and less modulated by higher-order ambiguity or social/contextual reinterpretation (e.g., the case of bird and wolf). As children age, they may increasingly rely on learned semantic frameworks or social knowledge, which could explain the decreasing consistency that was observed. This developmental shift may be understood through the lens of constructivist theories of cognitive development, which propose that early perceptual intuitions provide a foundation for later, more flexible – but potentially less consistent – conceptual reasoning (Piaget, 1952; Vygotsky, 1978). As children grow, their interpretations become increasingly shaped by experience, context and social learning, which may introduce greater variability in cross-modal associations despite more advanced cognitive capacities (see also Meltzoff & Borton, 1979; Mondloch & Maurer, 2004).

When interpreting the age effect, however, it is important to note that despite the high level of certainty that such an effect exists, we have found anecdotal evidence against the fact that it is meaningful (*BF* = 0.74 for Experiment 1; *BF* = 0.89 for Experiment 2). Additionally, contextual factors may have contributed to this effect. For example, since the majority of the children from the same grade were tested on the same day, external circumstances such as fatigue may have influenced their performance.

## Supplementary Information

Below is the link to the electronic supplementary material.

Supplementary file1 (DOCX 1.66 MB)

## Data Availability

Data available via the Open Science Framework at: https://osf.io/q2ug4.

## Footnotes

1 The bird is, of course, the only animal in the experimental stimuli whose vocalisations are typically described as ‘song’, namely birdsong.

2 We simplified the random-effects structure to include only random intercepts (1|id) in both models to reduce complexity and allow for a fair model comparison using LOO and WAIC.

3 However, it is important to note that the statistical modelling strategies employed in Di Stefano et al. (2025) and in the present study differ, reflecting the distinct nature of the data in each case. Specifically, Di Stefano and colleagues (2025) collected a continuous fit score for each audiovisual pairing on a 0–100 scale. In contrast, the present study recorded participants’ direct choices of images corresponding to each musical excerpt, resulting in a multinomial outcome variable. This method was preferred to facilitate 6- to 9-year-old participants in their choices, as forced-choice paradigms might be more appropriate for this age group (see, e.g., Li et al., 2025, for supporting evidence in children from age 10 years).

## Code availability

Will be made available upon request.
