# Supplementary material for: Auditory-conceptual associations in Peter and the Wolf and Carnival of the Animals: Evidence from 6- to 9-year-old children
Source: Psychon Bull Rev. 2026 Jan 5;33(1):28. doi: 10.3758/s13423-025-02804-4 (PMC12769493; doi:10.3758/s13423-025-02804-4)
Supplement: Supplementary file 1 — Supplementary file1 (DOCX 1.66 MB) [file 13423_2025_2804_MOESM1_ESM.docx]

SUPPLEMENTARY MATERIAL FOR:

Auditory-conceptual associations in *Peter and the Wolf* and *Carnival of the Animals*: Evidence from 6-9 year-old children

Di Stefano, N., Ansani, A., Focaroli, V., Borsella, R., Formenti, G., Velardi, A., Schiavio, A., Spence, C.

Journal: Psychonomic Bulletin & Review

Doi: 10.3758/s13423-025-02804-4

TableS1. Table of all the associations of *Peter and the Wolf.* The chance level is 20% and the chance region (ROPE) ranges between 15% and 20%. The percentage in ROPE (ROPE%) refers to the 89% Highest Density Interval (HDI) of each association’s posterior distribution.

| Music | Response | MAP | 89% LB | 89% UB | ROPE% |
| --- | --- | --- | --- | --- | --- |
| bird | bird | 85.77 | 76.02 | 93.54 | 0 |
| bird | cat | 2.28 | 0.51 | 6.95 | 0 |
| bird | duck | 1.13 | 0.12 | 5.39 | 0 |
| bird | grandpa | 5.36 | 1.7 | 12.68 | 0 |
| bird | wolf | 0.23 | 0 | 3.22 | 0 |
| cat | bird | 10.3 | 4.05 | 17.85 | 13.86 |
| cat | cat | 26.64 | 17.78 | 37.56 | 30.02 |
| cat | duck | 39.4 | 30.68 | 49.14 | 0 |
| cat | grandpa | 12.14 | 5.96 | 20.73 | 31.83 |
| cat | wolf | 6.35 | 1.89 | 13.14 | 0 |
| duck | bird | 4.19 | 0.8 | 10.77 | 0 |
| duck | cat | 22.6 | 14.29 | 33.18 | 60.51 |
| duck | duck | 44.58 | 35.25 | 54.22 | 0 |
| duck | grandpa | 9.55 | 3.79 | 17.32 | 12.85 |
| duck | wolf | 14.12 | 7.27 | 22.31 | 45.9 |
| grandpa | bird | 4.54 | 0.66 | 10.65 | 0 |
| grandpa | cat | 22.26 | 13.45 | 32.71 | 61.15 |
| grandpa | duck | 9.06 | 4.04 | 16.75 | 9.22 |
| grandpa | grandpa | 24.21 | 16.19 | 34.75 | 47.37 |
| grandpa | wolf | 33.96 | 24.49 | 44.02 | 0.47 |
| wolf | bird | 1.27 | 0 | 6.89 | 0 |
| wolf | cat | 10.04 | 4.43 | 18.45 | 18.25 |
| wolf | duck | 6.15 | 1.99 | 13.01 | 0 |
| wolf | grandpa | 12.22 | 5.62 | 19.8 | 28.45 |
| wolf | wolf | 64.22 | 54.17 | 74.31 | 0 |

TableS2. Table of all the associations of *The Carnival of the Animals.* The chance level is 14.28% and the chance region (ROPE) ranges between 10.71% and 17.85%. The percentage in ROPE (ROPE%) refers to the 89% Highest Density Interval (HDI) of each association’s posterior distribution.

| Music | Response | MAP | 89% LB | 89% UB | ROPE% |
| --- | --- | --- | --- | --- | --- |
| bird | bird | 9.01 | 4.22 | 17.13 | 44.1 |
| bird | chicken | 11.84 | 6.08 | 21.06 | 58.8 |
| bird | donkey | 13.19 | 8.21 | 20.42 | 71.91 |
| bird | elephant | 8.84 | 3.9 | 16.61 | 39.81 |
| bird | lion | 6.51 | 2.56 | 14.23 | 23.56 |
| bird | swan | 22.9 | 13.66 | 32.98 | 13.99 |
| bird | turtle | 17.13 | 9.53 | 27.22 | 46.75 |
| chicken | bird | 34.59 | 25.06 | 43.92 | 0 |
| chicken | chicken | 9.07 | 3.58 | 16.57 | 41.83 |
| chicken | donkey | 12.02 | 6.04 | 20.38 | 59.34 |
| chicken | elephant | 3.77 | 0.73 | 9.97 | 1.9 |
| chicken | lion | 11.4 | 5.11 | 19.33 | 55.36 |
| chicken | swan | 14.1 | 7.67 | 23.16 | 60.41 |
| chicken | turtle | 6.55 | 2.1 | 13.74 | 20.07 |
| donkey | bird | 16.51 | 9.16 | 25.58 | 51.97 |
| donkey | chicken | 11.71 | 5.44 | 20.08 | 57.5 |
| donkey | donkey | 16.45 | 9.5 | 26.54 | 49.74 |
| donkey | elephant | 14.54 | 7.32 | 22.58 | 61.25 |
| donkey | lion | 28.53 | 19.87 | 38.58 | 0 |
| donkey | swan | 4.11 | 0.65 | 10.58 | 4.61 |
| donkey | turtle | 0 | 0 | 2.26 | 0 |
| elephant | bird | 5.82 | 1.83 | 12.67 | 13.5 |
| elephant | chicken | 13.91 | 7.27 | 22.21 | 62.29 |
| elephant | donkey | 4.73 | 1.51 | 10.63 | 3.94 |
| elephant | elephant | 26.3 | 18.16 | 35.87 | 0 |
| elephant | lion | 11.66 | 5.18 | 18.77 | 55.85 |
| elephant | swan | 6.54 | 2 | 13.32 | 17.23 |
| elephant | turtle | 23.67 | 15.28 | 33.29 | 6.45 |
| lion | bird | 3.48 | 0.56 | 9.47 | 0.75 |
| lion | chicken | 10.9 | 5.01 | 19.11 | 56.15 |
| lion | donkey | 6.02 | 2.28 | 12.73 | 13.7 |
| lion | elephant | 16.94 | 9.16 | 24.97 | 55.01 |
| lion | lion | 39.07 | 29.51 | 49.64 | 0 |
| lion | swan | 11.3 | 5.5 | 19.5 | 58.27 |
| lion | turtle | 4.05 | 0.72 | 10.1 | 2.43 |
| swan | bird | 19.27 | 11.46 | 27.82 | 34.98 |
| swan | chicken | 1.79 | 0.01 | 6.54 | 0 |
| swan | donkey | 9.52 | 4.35 | 17.37 | 46.64 |
| swan | elephant | 3.64 | 0.66 | 9.7 | 1.48 |
| swan | lion | 0 | 0 | 2.21 | 0 |
| swan | swan | 41.91 | 31.75 | 52 | 0 |
| swan | turtle | 16.75 | 9.35 | 25.6 | 52.34 |
| turtle | bird | 16.4 | 8.96 | 25.22 | 53.73 |
| turtle | chicken | 9.47 | 3.46 | 16.38 | 40.17 |
| turtle | donkey | 8.16 | 3.7 | 15.87 | 36.43 |
| turtle | elephant | 16.01 | 8.84 | 24.92 | 54.08 |
| turtle | lion | 8 | 3.51 | 16.2 | 37.69 |
| turtle | swan | 18.95 | 10.72 | 28.04 | 34.31 |
| turtle | turtle | 14.28 | 7.3 | 22.93 | 59.87 |


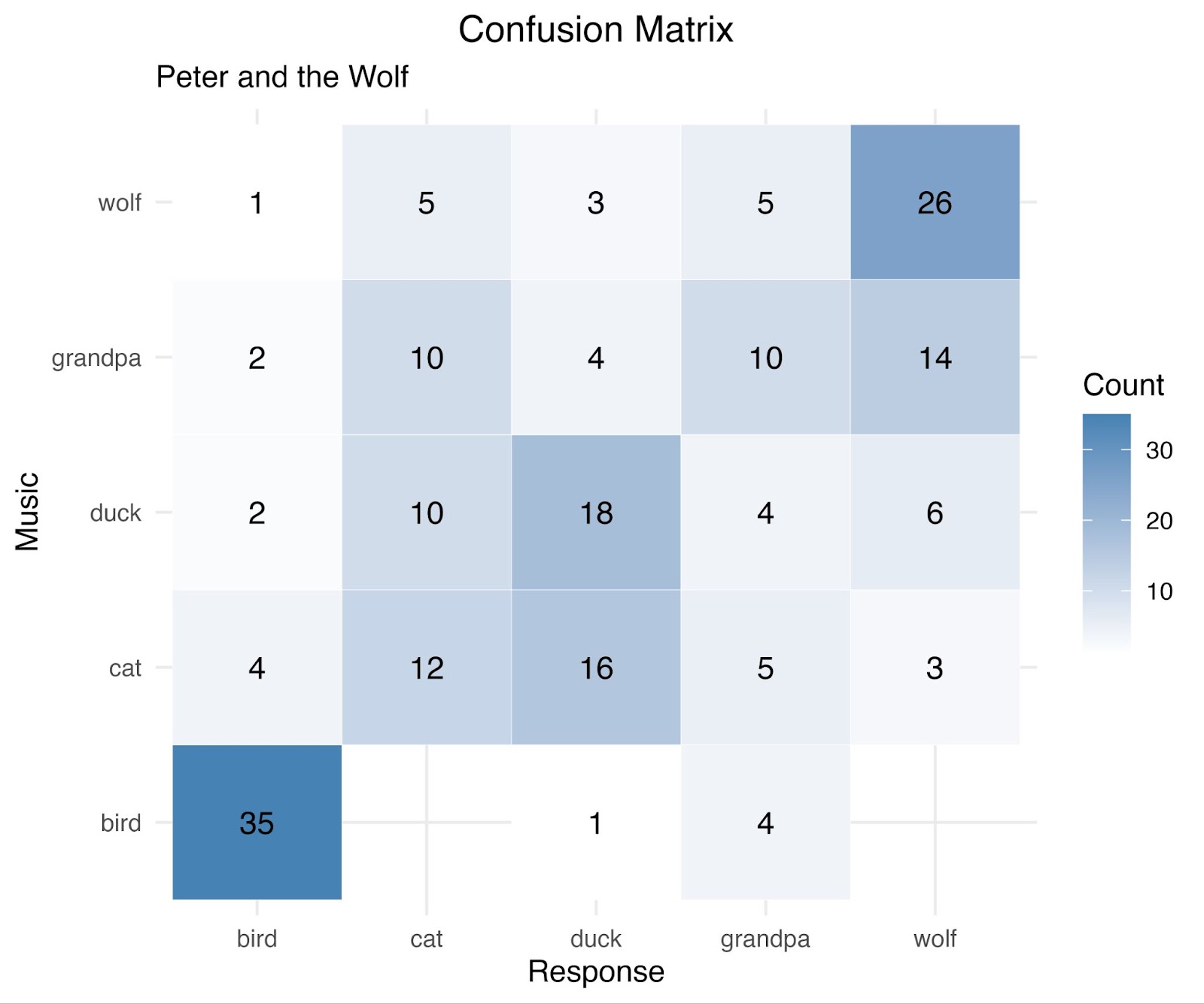


Figure S1. Confusion matrix from Experiment 1. The values represent the frequency with which participants selected each specific association.

**
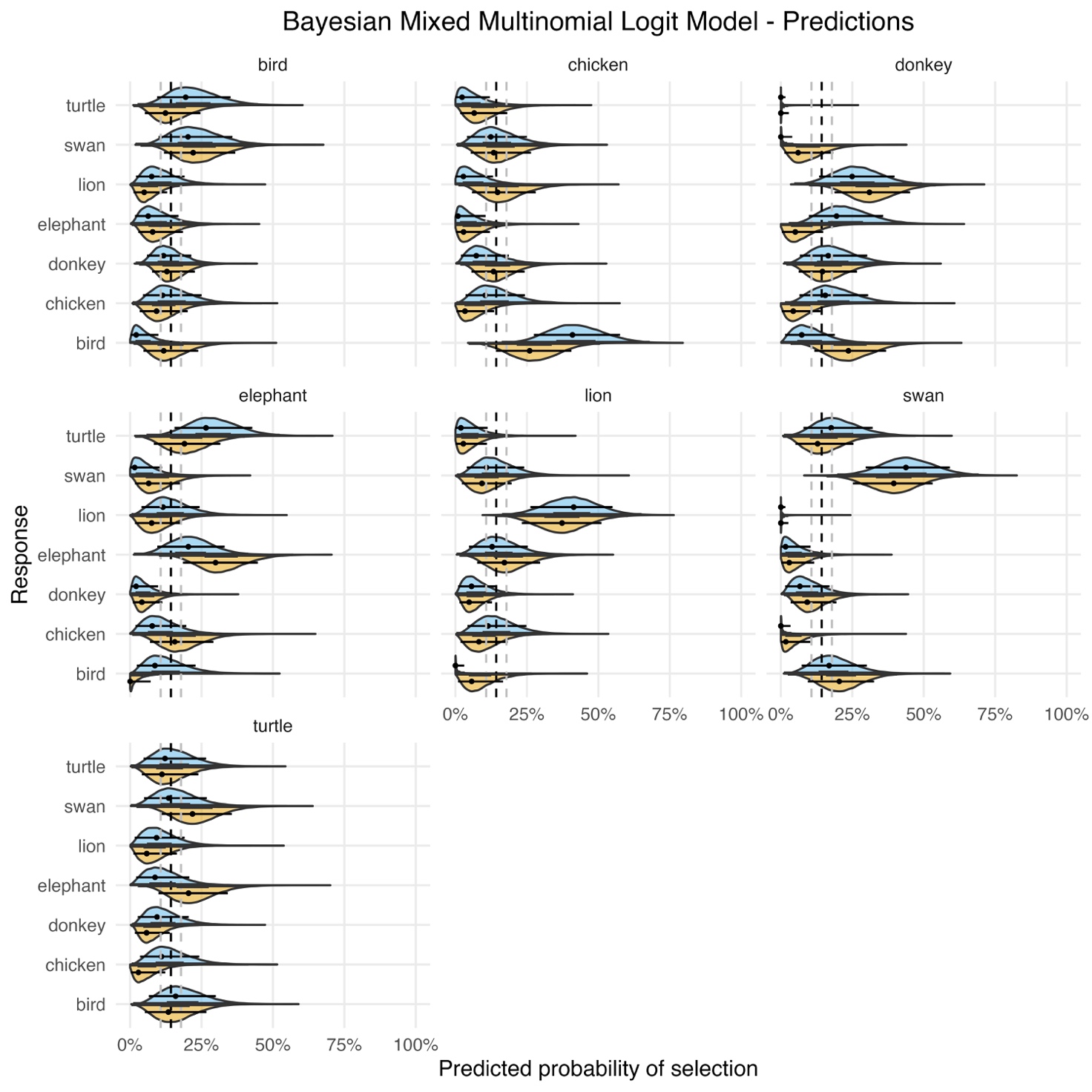
**

Figure S2. Predictions of the Bayesian Mixed Multinomial Logit Model. The label on top of each panel represents the musical excerpts. On the x-axis, the posterior distributions of the predicted probabilities of the image choices are plotted. On the y-axis are the participants’ responses (i.e., images). The area delimited by the grey dashed lines represents the ROPE. The horizontal black lines represent the 89% credible interval, whereas the middle point indicates the MAP.


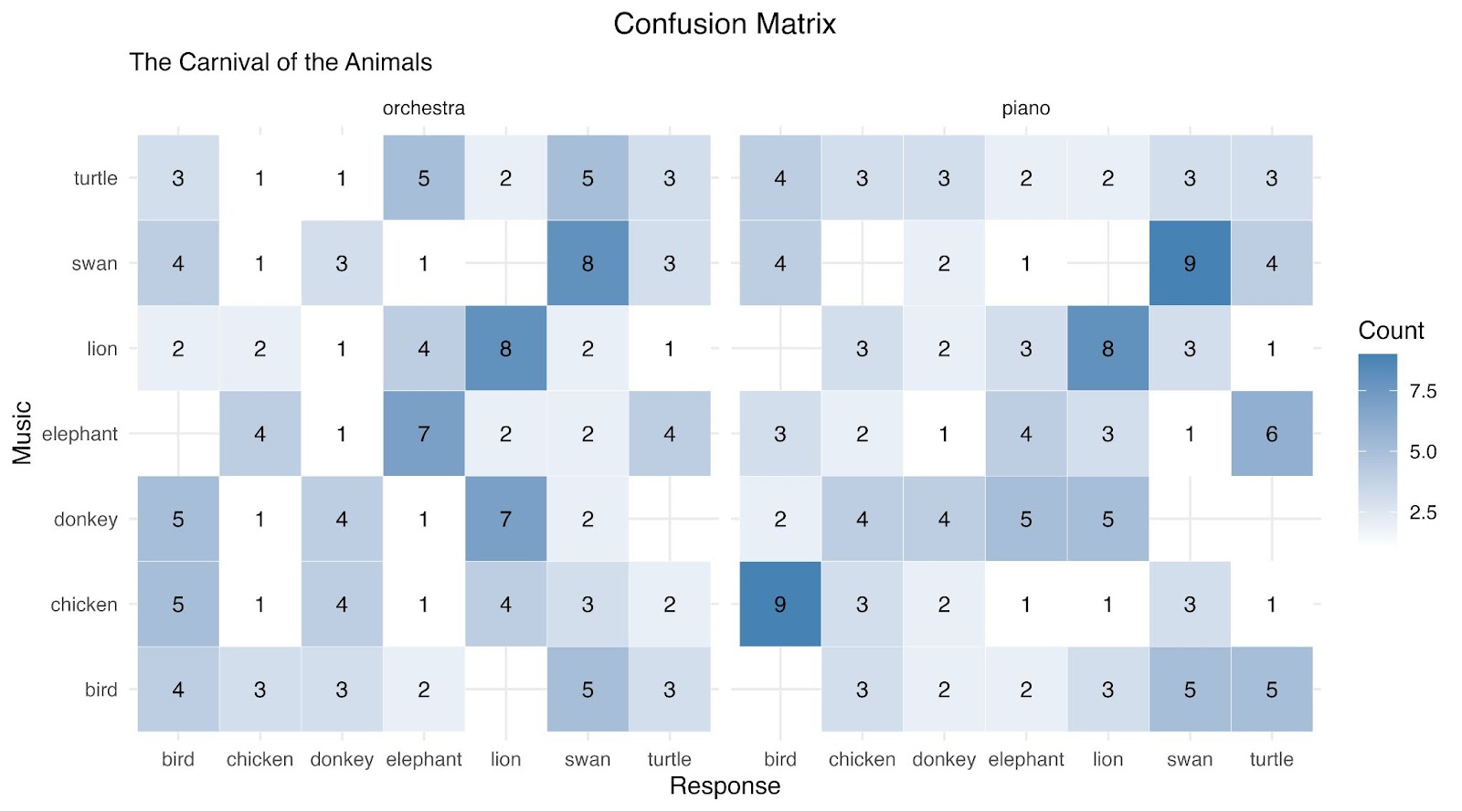


Figure S3. Confusion matrix from Experiment 2. The values represent the frequency with which participants selected each specific association.

Figure S4. Visual stimuli used in Experiment 1.

Figure S5. Visual stimuli used in Experiment 2.
